# Non-human primate to human immunobridging demonstrates a protective effect of Ad26.ZEBOV, MVA-BN-Filo vaccine against Ebola

**DOI:** 10.1038/s41541-022-00564-z

**Published:** 2022-11-30

**Authors:** Viki Bockstal, Maarten Leyssen, Dirk Heerwegh, Bart Spiessens, Cynthia Robinson, Jeroen N. Stoop, Ramon Roozendaal, Thierry Van Effelterre, Auguste Gaddah, Griet A. Van Roey, Laura Solforosi, Roland Zahn, Benoit Callendret, Jenny Hendriks, Kerstin Luhn, Macaya Douoguih, Hanneke Schuitemaker, Johan Van Hoof

**Affiliations:** 1 Janssen Vaccines & Prevention B.V., Leiden, The Netherlands; 2 Janssen Research and Development, Beerse, Belgium; 3 Present Address: ExeVir, Ghent, Belgium; 4 Present Address: University of Leuven, Leuven, Belgium

**Keywords:** Viral infection, Biomarkers

## Abstract

Without clinical efficacy data, vaccine protective effect may be extrapolated from animals to humans using an immunologic marker that correlates with protection in animals. This immunobridging approach was used for the two-dose Ebola vaccine regimen Ad26.ZEBOV, MVA-BN-Filo. Ebola virus (EBOV) glycoprotein binding antibody data obtained from 764 vaccinated healthy adults in five clinical studies (NCT02416453, NCT02564523, NCT02509494, NCT02543567, NCT02543268) were used to calculate mean predicted survival probability (with preplanned 95% confidence interval [CI]). We used a logistic regression model based on EBOV glycoprotein binding antibody responses in vaccinated non-human primates (NHPs) and NHP survival after EBOV challenge. While the protective effect of the vaccine regimen in humans can be inferred in this fashion, the extrapolated survival probability cannot be directly translated into vaccine efficacy. The primary immunobridging analysis evaluated the lower limit of the CI against predefined success criterion of 20% and passed with mean predicted survival probability of 53.4% (95% CI: 36.7–67.4).

## Introduction

Since the discovery of the Ebola virus (EBOV) in 1976^1^, the number of outbreaks and fatalities have been increasing over time, with the devastating 2014 to 2016 outbreak in West Africa causing more cases and deaths than all previous outbreaks combined^2^. Since then, six EBOV disease (EVD) outbreaks have been declared in the Democratic Republic of the Congo (DRC) and Guinea^3^.

Reactive use of rVSV-ZEBOV-GP (Ervebo^®^), a recombinant replicating vector-based vaccine that demonstrated high efficacy during the outbreaks in Guinea^4^ and DRC^5^, is recommended by the World Health Organization (WHO) Strategic Advisory Group of Experts on Immunization (SAGE) during outbreak control for people at high risk of EVD^6^. Despite the demonstrated efficacy of this vaccine in a reactive setting, the long-lasting 2018 to 2020 outbreak highlighted the importance of complementary prophylactic vaccines. Indeed, the WHO SAGE recommended vaccination of lower-risk populations with the two-dose Ad26.ZEBOV, MVA-BN-Filo regimen (Janssen Vaccines and Prevention, B. V.) in the 2018 to 2020 outbreak^6^. Ad26.ZEBOV is a monovalent, recombinant, replication-incompetent, Ad26-vectored vaccine encoding the glycoprotein (GP) of the EBOV Mayinga variant. MVA-BN-Filo (Janssen Vaccines and Prevention, B.V., produced in collaboration with Bavarian Nordic) is a recombinant, non-replicating, modified vaccinia Ankara (MVA)–vectored vaccine encoding the EBOV Mayinga variant GP, the Sudan virus Gulu GP, the Marburg virus Musoke GP, and the Taï Forest virus nucleoprotein. Furthermore, conditional approval under an exceptional emergency situation was granted by the Rwanda Food and Drug Authority, and the regimen was used in a large vaccination campaign^7^. In July 2020, the Ad26.ZEBOV, MVA-BN-Filo regimen received approval for prophylactic use in adults and children aged ≥1 year in the European Union (Zabdeno^®^, Mvabea^®^)^8,9^.

Specific regulatory guidelines, including the U.S. Food and Drug Administration (FDA) Animal Rule^10^ and European Medicines Agency (EMA) conditional approval^11^ or approval under exceptional circumstances^12^, allow for licensure when human efficacy studies are not feasible. These guidelines specify that an immunologic marker that correlates with protection in a suitable animal model can be used to demonstrate the likelihood of clinical benefit, a concept called immunobridging, as a basis for licensure, with additional post-licensure commitments^10–12^. Evaluation of the protective effect of Ad26.ZEBOV, MVA-BN-Filo for European licensure under exceptional circumstances was based on such an immunobridging approach. A non-human primate (NHP) challenge model was used in which disease symptoms after EBOV challenge are similar to, but more stringent than, human EVD^13,14^. The NHP model is considered stringent as it is fully lethal, compared to an average human case fatality rate of 50%, and NHPs have a shorter time to onset of symptoms (average of 5.4 days compared to 6.2-9.7 days in humans) and an extremely rapid disease progression with a shorter time to death (after symptom onset, mean survival time in NHPs is 1.4 days relative to 5.8-14.4 days to death for lethal human cases)^15–24^. It has long been recognized that EBOV GP binding antibody levels correlate with vaccine-induced protection against EBOV^25,26^, which we confirmed for the Ad26.ZEBOV, MVA-BN-Filo vaccine regimen in Roozendaal et al.^24^. Based on this, Roozendaal et al. constructed a logistic regression model that relates binding antibody levels measured in NHPs to their survival probability^24^. For the current immunobridging analysis, we utilized a “one lab, one assay” approach and rebuilt the logistic regression model using GP binding antibody concentrations that were measured by the same accredited laboratory where the clinical samples were analyzed. We describe the modeling of antibody levels after vaccination in participants from five clinical studies to calculate a mean predicted survival probability with an associated confidence interval (CI), of which the lower limit had a pre-defined success criterion. Due to the stringency of the NHP model, the calculated mean predicted survival probability is expected to be an underestimation of clinical vaccine efficacy. Hence, immunobridging provides support for the vaccine protective effect in humans, yet the inferred mean predicted survival probability cannot be directly translated into actual vaccine efficacy.

## Results

### Protective effect in healthy adults (aged 18-50 years)

The primary immunobridging analysis was based on the pooled per-protocol immunogenicity (PPI) dataset of 764 healthy adults from five studies (EBL2001 [France, UK]^27,28^, EBL2002 [Burkina Faso, Côte d’Ivoire, Kenya, Uganda]^29–31^, EBL3001 [Sierra Leone]^32–34^, EBL3002 [USA]^35,36^, and EBL3003 [USA]^36,37^). The baseline and demographic characteristics are summarized in Supplementary Table 1 in the Supplementary Appendix. The mean age was 30.6 years, and most participants were male (65%) and from either the USA (51%) or African countries (43% in total; 28% from Sierra Leone). At 21 days post-Dose 2, the EBOV GP binding antibody geometric mean concentration (GMC) was 3918 enzyme-linked immunosorbent assay (ELISA) units (EU)/mL for study EBL3001 conducted in Sierra Leone and ranged from 8109 EU/mL to 11,054 EU/mL in the other four clinical studies (conducted in Burkina Faso, Côte d’Ivoire, France, Kenya, Uganda, UK, and USA); regardless of GMC, responder rates were consistent in all five studies, ranging from 98% to 100% (Supplementary Table 2 in the Supplementary Appendix).

The primary immunobridging analysis demonstrated a mean predicted survival probability of 53.4% with a lower limit of the pre-planned 95% CI of 36.7% and a post-hoc 98.7% CI (a conservative threshold regularly used in interim analyses) with a lower limit of 33.8% (Table 1). Both lower limits were well above 20%, thereby passing the pre-defined success criterion of the primary immunobridging analysis. Although the mean predicted survival probability cannot be interpreted as the actual vaccine efficacy, the analysis provides support for a protective effect of the vaccine regimen in humans.

**Table 1:** Immunobridging analysis using a logistic regression model based on data from NHPs vaccinated with the Ad26.ZEBOV, MVA-BN-Filo vaccine regimen in a 56-day interval; PPI analysis set.

Participants Vaccinated, N	764
Pre-planned Immunobridging Analysis	
Mean Predicted Survival Probability, % (95% CI)	53.4 (36.7–67.4)
Post-hoc Analysis	
Mean Predicted Survival Probability, % (98.7% CI)	53.4 (33.8–70.9)
* O’Brien-Fleming Adjustment (One-sided Alpha of 0.0066)*	

*CI* confidence interval, *NHP* non-human primate, *PPI* per-protocol immunogenicity.

This analysis was based on the pooled data of healthy adults (aged 18-50 years) vaccinated with Ad26.ZEBOV, MVA-BN-Filo in a 56-day interval in five clinical studies (EBL2001, EBL2002, EBL3001, EBL3002, and EBL3003) using a logistic regression model based on NHP data from the Ad26.ZEBOV, MVA-BN-Filo vaccine regimen in a 56-day interval.

**Fig. 1:**
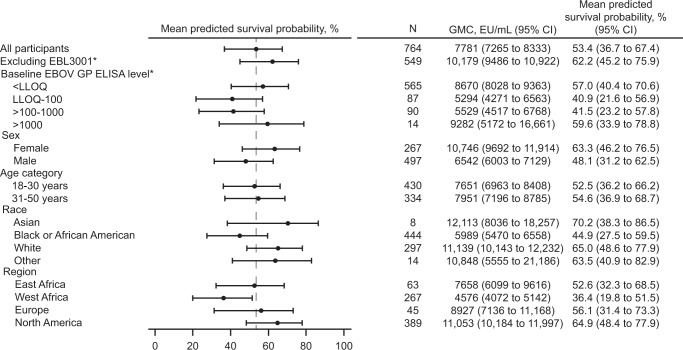
Forest plot of mean predicted survival probability and 95% CI – prespecified subgroup analyses by baseline EBOV GP binding antibody concentration, sex, age category, race, and region; PPI analysis set. This analysis was based on the pooled data of healthy adults (aged 18-50 years) vaccinated with Ad26.ZEBOV, MVA-BN-Filo in a 56-day interval in five clinical studies (EBL2001, EBL2002, EBL3001, EBL3002, and EBL3003) using a logistic regression model based on NHP data from the Ad26.ZEBOV, MVA-BN-Filo vaccine regimen in a 56-day interval. Mean predicted survival probability and the 95% bootstrapped CI are reported. CI confidence interval, EBOV Ebola virus, ELISA enzyme-linked immunosorbent assay, EU enzyme-linked immunosorbent assay units, GMC geometric mean concentration, GP glycoprotein, LLOQ lower limit of quantification (36.11 EU/mL), N number of participants with data, NHP non-human primate, PPI per-protocol immunogenicity, vertical dashed line = mean predicted survival probability from primary analysis including all participants. *The subgroup analyses excluding the participants from clinical study EBL3001 and the stratification by baseline EBOV GP ELISA level are described in more detail in the Supplementary Results.

**Fig. 2:**
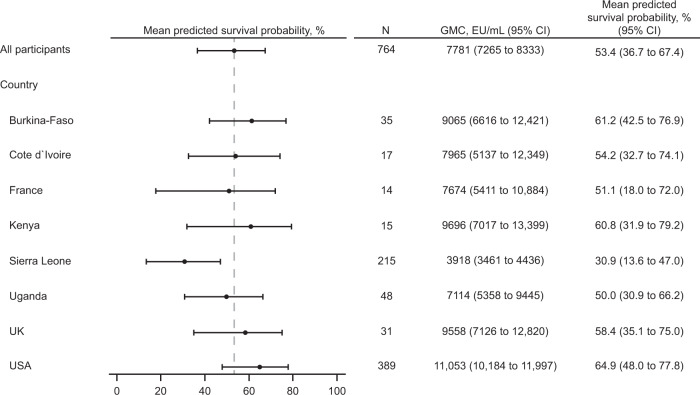
Forest plot of mean predicted survival probability and 95% CI – post hoc subgroup analyses by country. This analysis was based on the pooled data of healthy adults (aged 18-50 years) vaccinated with Ad26.ZEBOV, MVA-BN-Filo in a 56-day interval in five clinical studies (EBL2001, EBL2002, EBL3001, EBL3002, and EBL3003) using a logistic regression model based on NHP data from the Ad26.ZEBOV, MVA-BN-Filo vaccine regimen in a 56-day interval. Mean predicted survival probability and the 95% bootstrapped CI are reported. CI confidence interval, EU enzyme-linked immunosorbent assay units, GMC geometric mean concentration, N number of participants with data, NHP non-human primate; vertical dashed line = mean predicted survival probability from primary analysis including all participants.

Sensitivity to potential influencing factors is shown in Fig. 1. Given the inherent variability of subgroup analyses, and that some subgroups were very small and therefore not sufficiently powered, the lower limit of the CI was not formally compared against the success criterion defined for the primary analysis. The pre-specified sensitivity analyses were consistent overall with the primary analysis, and for the subgroup analyses by region, only a lower mean predicted survival probability was observed in the West African subgroup (36.4%), containing pooled data from Burkina Faso, Côte d’Ivoire, and Sierra Leone. A post-hoc analysis performed by country (Fig. 2) indicated that, in line with the lower immunogenicity observed in study EBL3001 (Supplementary Table 2 in the Supplementary Appendix), a lower country-specific mean predicted survival probability was obtained for Sierra Leone (30.9%), but not for the other West African countries Burkina Faso (61.2%) and Côte d’Ivoire (54.2%).

### Protective effect in specific subpopulations: healthy adults (aged > 50 years), people living with human immunodeficiency virus (PLWH; aged 18-50 years), and children (aged 1-17 years)

**Fig. 3:**
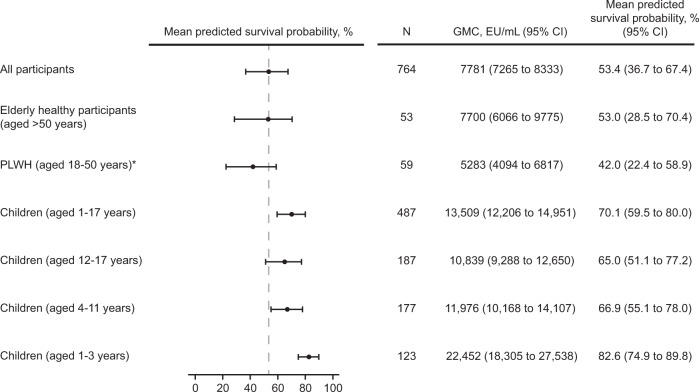
Forest plot of mean predicted survival probability and 95% CI for healthy adults aged >50 years, PLWH (aged 18-50 years), and children (aged 1-17 years); PPI analysis set. This analysis was based on the pooled data of participants vaccinated with Ad26.ZEBOV, MVA-BN-Filo in a 56-day interval in five clinical studies (EBL2001, EBL2002, EBL3001, EBL3002, and EBL3003) using a logistic regression model based on data from NHPs vaccinated with the Ad26.ZEBOV, MVA-BN-Filo vaccine regimen in a 56-day interval. CI confidence interval, EU enzyme-linked immunosorbent assay units, NHP non-human primate, PLWH people living with human immunodeficiency virus, PPI per-protocol immunogenicity. *PLWH were on a stable antiretroviral therapy regimen, had a CD4 + cell count >350 cells/μL, and were considered to be in otherwise reasonably good medical condition.

Subgroup analyses were performed to assess the protective effects in older healthy adults, adult PLWH, and children because immune responses may vary in different populations and age groups. Among the 53 older participants aged >50 years, the mean age was 57.1 years (standard deviation, 4.6) and ages ranged from 51 to 69 years. The antibody GMC at 21 days post-Dose 2 was 7700 EU/mL in older adults, 5283 EU/mL in PLWH, and 13,509 EU/mL in children aged 1-17 years, with corresponding responder rates of 98%, 100%, and 98.5%, respectively (Fig. 3 and Supplementary Table 3 in the Supplementary Appendix). The mean predicted survival probability was 53% in older adults, 42.0% in PLWH, and 70.1% in children aged 1-17 years (Fig. 3). Among children, the mean predicted survival probability increased with decreasing age groups: 65.0% in children aged 12-17 years, 66.9% in children aged 4-11 years, and 82.6% in children aged 1-3 years (Fig. 3). Overall, these analyses, as well as similar analyses conducted in the full analysis set (FAS; Supplementary Table 4 in the Supplementary Appendix) indicate that Ad26.ZEBOV, MVA-BN-Filo is likely to confer protection against EVD in elderly healthy adults, PLWH, and children.

## Discussion

The EMA and FDA guidelines on the clinical evaluation of vaccines state that when it is not possible to conduct a traditional clinical efficacy study, other approaches to estimate vaccine efficacy can be considered, including extrapolation of vaccine efficacy from animals to humans^10–12^. While this assumes that the protective mechanism is conserved between the animal model and humans, the immunologic parameter selected for immunobridging only needs to correlate with survival and is not necessarily the main mechanistic contributor to protection. We previously identified EBOV GP binding antibodies as an immune parameter strongly correlating with survival after EBOV challenge in vaccinated NHPs^24^. Vaccination-elicited immune responses are similar between NHPs and humans, and the same assay in the same laboratory was used to measure the vaccine-elicited binding antibody responses in NHPs and humans^24^. An immunobridging approach based on EBOV GP binding antibody responses was used to infer the protective effect of the vaccine regimen in people. A 3-week post–second vaccination time point was selected based on the kinetics of the antibody response observed in NHPs and confirmed in humans^38–40^; it was the final immunogenicity sampling time point before the transfer of NHPs to the BSL4 facility for challenge one week later. At this time point and at the clinical dose, human antibody response levels are typically two- to four-fold lower than antibody responses in NHPs^28,30,33,36,38–40^. While NHPs are protected at this dose level, the predicted survival probability based on human immune response levels is lower. However, as noted previously, the survival rate for EVD in humans is higher than that of NHPs. As no adjustments were made for this in the immunobridging, lower antibody levels do not necessarily indicate lower survival.

Prior to unblinding of the five clinical studies, it was agreed with the EMA that the vaccine would be considered protective if the lower limit of the 95% CI around the mean predicted survival probability was above a pre-specified success criterion of 20%. This success criterion was selected considering that, in unvaccinated NHPs, the observed survival was 0% (0/13 unvaccinated NHPs survived). A lower limit of 20% for the 95% CI provides a sufficiently large margin to indicate a true protective effect, and is similar to thresholds employed in vaccine field efficacy studies supporting regulatory approval^41^. Due to the stringency of the NHP challenge model, the calculated mean predicted survival probability is difficult to interpret and the inferred likelihood of protection is likely an underestimation of clinical vaccine efficacy. The primary immunobridging analysis demonstrated a mean predicted survival probability of 53.4%. Lower limits of the pre-planned 95% CI and post-hoc 98.7% CI, 36.7% and 33.8%, respectively, were well above the pre-specified success criterion of 20%, thereby confirming the likelihood of protection in healthy adults (aged 18-50 years). While immunobridging provides support for the vaccine protective effect, the mean predicted survival probability derived from the model cannot be directly translated into the actual level of clinical vaccine efficacy, which will need to be determined in a field study. In view of the severity and lethal risk posed by EVD, the benefit of likely protection together with the absence of evidence of a safety concern^27–37^ outweighs the current uncertainty on the exact clinical vaccine efficacy.

A substantial number of healthy adults (120 out of 899) included in the FAS had received a delayed MVA-BN-Filo vaccination (with an interval up to 454 days between the two vaccines) due to a study pause. As reported previously, EBOV GP binding antibody responses in participants who received a delayed second dose were at least as high as those who received the two vaccines in the per-protocol–defined 56-day interval^28,30,31^, demonstrating that the vaccine regimen is at least as immunogenic if the second vaccination is administered later than planned. In line with this observation, no notable differences were observed between the immunobridging analyses based on the FAS (see Supplementary Notes) and the primary (PPI) analysis set. The predictive vaccine protective effect was inferred in healthy adults aged >50 years, PLWH, and children aged ≥1 year, with the highest survival estimate (82.6%) observed in the youngest children (aged 1-3 years) in a post-hoc analysis.

The lower country-specific mean predicted survival probability calculated for Sierra Leone (30.9%) was expected based on the lower EBOV GP binding antibody GMC observed 21 days post-MVA-BN-Filo in study EBL3001 (Supplementary Table 2 in the Supplementary Appendix). This lower immunogenicity was not related to pre-existing immunity against the Ad26 vector, nor to demographic or logistic factors (shipment and storage of vaccines and/or sera). The lower immunogenicity in Sierra Leone may be explained by a combination of factors specific to the region as the study was conducted in the Kambia district, one of the most rural areas of Sierra Leone^32,33^. Individuals living in rural areas compared to more urban areas have lower life expectancy and worse health status^42^, which may contribute to lower immune responses to vaccination. Other geographic differences, such as nutritional deficiencies or genetic variability, could also affect vaccine immunogenicity^43,44^. Even in the worst-case scenario of a one-to-one translation of mean predicted survival probability to vaccine efficacy, epidemiologic modeling predicts that, at a population level, 30% vaccine efficacy would still result in a significant reduction in deaths caused by EVD (dependent on vaccination coverage)^45^. Upon administration of an Ad26.ZEBOV booster two years after the primary regimen, participants from Sierra Leone showed a strong anamnestic response within seven days^33^, demonstrating that Ad26.ZEBOV, MVA-BN-Filo had induced immunologic memory that likely contributes to protection upon exposure to EBOV.

This immunobridging analysis uses a logistic regression curve based only on the level of virus-specific antibodies present in the circulation shortly after vaccination and, while this does not take waning immunity after vaccination into account, it also does not allow vaccination-induced immunologic memory to contribute to the calculated mean predicted survival probability. In addition, due to the short time to death in NHPs, the contribution of immune memory to the durability of vaccine-elicited protection cannot be measured. Assessing the durability of the vaccine protective effect would require an animal model in which the disease course is slower and more reflective of human disease^24^, allowing the vaccination-induced immunologic memory to contribute to protection. However, such a model is currently not available and, in the absence of evidence for durability of protection, an Ad26.ZEBOV booster after the primary Ad26.ZEBOV, MVA-BN-Filo vaccination regimen is currently recommended as a precautionary measure in the situation of imminent risk of exposure to maximize the protective effect^8,9^. The research described here supports a new approach to demonstrate the probability of vaccine-mediated protection. Immunobridging has been used to support regulatory approval of the anthrax vaccine (BioThrax^®^)^46^ and will likely be used more often for other emerging infectious diseases, like Marburg virus disease, Crimean-Congo hemorrhagic fever, or Nipah^47^, for which evaluation of clinical efficacy testing will be as challenging.

## Methods

### Regression model

The logistic regression model used for this immunobridging study was previously developed using data from NHP challenge studies^24^. Seven NHP challenge studies were conducted with a single 0.5 mL intramuscular administration of the well-characterized EBOV Kikwit virus recommended by the Filovirus Animal Non-Clinical Group (FANG)^24,48^ at a fully lethal target dose of 100 plaque-forming units. A penalized logistic regression model based on NHP data was developed using Firth’s method^49^, with survival outcome as a dependent variable and EBOV GP binding antibody concentrations (EU/mL, log_10_) measured 21 days post-Dose 2 as an independent variable. For the model described in Roozendaal et al.^24^, EBOV GP binding concentrations were analyzed by the Battelle Biomedical Research Center (Columbus, OH, USA). For the current immunobridging analysis, all NHP samples were reanalyzed by Q^2^ Solutions Vaccine Testing Laboratory (San Juan Capistrano, CA, USA) to enable a direct comparison to the human immunogenicity data. More information on the selection of the immune parameter and the model development can be found in Roozendaal et al.^24^.

The immunogenicity data of participants vaccinated with the two-dose, heterologous Ad26.ZEBOV, MVA-BN-Filo regimen in a 56-day interval were obtained from two phase 2 and three phase 3 randomized, observer-blind, and placebo-controlled studies in healthy adults, PLWH, and pediatric (aged 1-17 years) participants: EBL2001 (France, UK)^27,28^, EBL2002 (Burkina Faso, Côte d’Ivoire, Kenya, Uganda)^29–31^, EBL3001 (Sierra Leone)^32–34^, EBL3002 (USA)^35,36^, and EBL3003 (USA)^36,37^. All participants received an intramuscular injection (0.5 mL) with 5 × 10^10^ virus particles of Ad26.ZEBOV as Dose 1, followed 56 days later by 1 × 10^8^ Infectious Units of MVA-BN-Filo as Dose 2 (heterologous two-dose Ad26.ZEBOV, MVA-BN-Filo vaccine regimen). The protocol-defined window around Dose 2 was ±3 days for all studies, except EBL2001 (±1 day) and EBL3001 (±7 days). In all five clinical studies, EBOV GP binding antibody concentrations were measured by Q^2^ Solutions Laboratory. Study details are available online^27,29,32,35,37^ and are previously published^28,30,31,33,34,36^.

For immunogenicity assessments in the previous NHP challenge studies and clinical studies, serum samples were obtained from all NHPs and clinical study participants immediately before the first vaccination and three weeks after the second vaccination. Immunoglobulin G responses against EBOV GP were analyzed in all NHP and clinical samples using the same EBOV GP (Kikwit) FANG ELISA^28,50^, validated for both human and NHP serum, in the same Q^2^ Solutions Laboratory. Responses were summarized as group GMCs with 95% CI. For human samples, all values below the lower limit of quantification (LLOQ [36.11 EU/mL]) were imputed with half of the LLOQ value (ie, LLOQ/2). Clinical study participants were considered responders if the post-vaccination concentration was >2.5-fold the LLOQ in baseline seronegative individuals or >2.5-fold the baseline value in baseline seropositive participants.

**Fig. 4:**
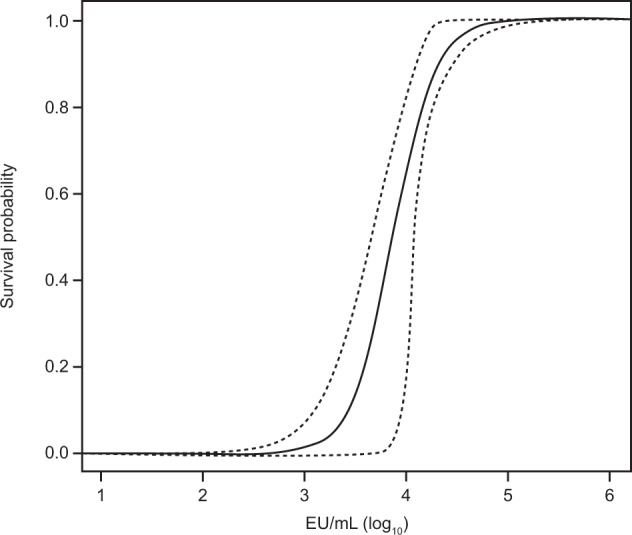
Logistic regression model for main regimen. Figure depicts the logistic regression model and its 95% confidence band (bootstrap-derived using 10,000 bootstraps of the NHP data of the main regimen). EU enzyme-linked immunosorbent assay units, NHP non-human primate.

Two logistic regression models were constructed for the immunobridging analysis. The primary analysis model shown in Fig. 4 contained only data from NHPs vaccinated with Ad26.ZEBOV, MVA-BN-Filo in a 56-day interval (*N* = 66). The second model was based on all available data of NHPs (*N* = 108) vaccinated with either Ad26.ZEBOV or Ad26.Filo and MVA-BN-Filo in different vaccine sequences and intervals between doses. The second model was only used in a sensitivity analysis to evaluate the robustness of the primary immunobridging result. The data that were used for the calculation of the logistic regression models are shown in Supplementary Table 5 in the Supplementary Appendix, and the result is shown in Supplementary Table 6.

The fitted logistic regression model was used to predict a survival probability for each human ELISA value measured at 21 days post-Dose 2. The individual predicted human survival probabilities were averaged to calculate the mean predicted survival probability. First, a 95% CI was calculated using a non-parametric double bootstrap method. This method consisted of resampling the NHP and human datasets 10,000 times with replacement, repeating the fitting of a logistic model on the re-sampled NHP data, and calculating a mean predicted survival probability by inserting the re-sampled human ELISA data into the logistic model and averaging the predicted individual survival probabilities. As a result, 10,000 mean predicted survival probabilities were obtained. The 95% CI was derived as the 2.5th and 97.5th percentiles of the distribution of the mean predicted survival probabilities.

The immunobridging analysis using data from the five clinical studies was originally pre-planned as an interim futility analysis, with no foreseen Type I error rate adjustment. In view of the 2018 to 2020 outbreak and the persisting public health need, this analysis was used as the basis for marketing authorization approval in Europe. To adjust the CI for alpha spending post-hoc, a 98.7% CI was calculated. The 98.7% CI was based on the O’Brien-Fleming alpha spending rules, as this approach is conservative and regularly used in interim analyses (approximately 65% of the pre-planned data available, resulting in an O’Brien-Fleming adjusted one-sided alpha of 0.0066 [obtained using Wang-Tsiatis bounds where Δ = 0]). The same non-parametric double bootstrap procedure was used to calculate the 98.7% CI but was based on 100,000 bootstraps to ensure sufficient resolution in the extreme regions of the distribution.

### Statistical analyses

The primary analysis aimed to evaluate whether the lower limit of the CI was above a pre-defined success criterion of 20%, a cutoff agreed upon with the EMA. Immunogenicity data from healthy adult participants (aged 18-50 years) vaccinated with Ad26.ZEBOV, MVA-BN-Filo in the five contributing clinical studies were pooled. The FAS comprised all participants who were randomized (and non-randomized, open-label stage 1 of study EBL3001) and received ≥1 dose of study vaccine, regardless of the occurrence of protocol deviations. The PPI analysis set represented the primary analysis set and included all randomized (and non-randomized, open-label stage 1 of study EBL3001) and vaccinated participants who received Dose 1 and Dose 2 within the protocol-defined windows, had ≥1 evaluable post-vaccination immunogenicity sample, and had no major protocol deviations influencing the immune response. Only participants with an available 21 days post-Dose 2 ELISA result were included in the immunogenicity and immunobridging analyses. Immunogenicity data were analyzed descriptively, and immunobridging was performed on the PPI analysis set (primary analysis) and on the FAS.

### Immunobridging sensitivity analyses

Pre-specified immunobridging sensitivity analyses were conducted to evaluate the robustness of the primary analysis and assess potential influencing factors, such as baseline positivity in the ELISA, sex, age, race, and geographic region (Supplementary Tables 1, 7, and 8 in the Supplementary Appendix). Firstly, because Sierra Leone was the only country included in the five clinical studies that was previously affected by an EBOV outbreak, the analysis was repeated including only participants from study EBL3001 (which was conducted in Sierra Leone), stratified per baseline EBOV GP FANG ELISA levels (<LLOQ [36.11 EU/mL], LLOQ-100, >100-1000, >1000 EU/mL), to assess the impact of pre-existing EBOV GP binding antibody levels on the immunobridging analysis (Supplementary Fig. 1 in the Supplementary Appendix). Secondly, to further assess the effect of possible pre-exposure to EBOV, the analysis was repeated, excluding the participants of the Sierra Leone study (EBL3001; Supplementary Fig. 2)^32^. Thirdly, demographic subanalyses were conducted, stratified by age (18-30 and 31-50 years of age), sex (female and male), race (Asian, Black or African American, White, and other), and geographic region (East Africa [Kenya, Uganda], West Africa [Burkina Faso, Côte d’Ivoire, Sierra Leone], Europe [France, UK], and North America [USA]; Fig. 1 and Supplementary Fig. 2). Mean predicted survival probabilities with a 95% CI were also calculated based on the pooled data of healthy elderly participants (aged > 50 years), PLWH (aged 18-50 years), and children (1-17 years, and in three age categories: 1-3 years, 4-11 years, and 12-17 years) using the primary analysis model (Fig. 3). PLWH in this analysis were on a stable antiretroviral therapy regimen, had a CD4 + cell count >350 cells/μL, and were considered to be in otherwise reasonably good medical condition without an acquired immunodeficiency syndrome–defined diagnosis or a clinically significant disease. For all sensitivity analyses, the 95% CI were calculated based on the non-parametric double bootstrap method. Fourthly, post-hoc, a subgroup immunobridging analysis was conducted by country (Burkina Faso, Côte d’Ivoire, France, Kenya, Sierra Leone, Uganda, UK, and USA), with the 95% CI calculated based on the non-parametric double bootstrap method (Fig. 2). Finally, as a prespecified sensitivity analysis, the primary analysis was repeated using the second model based on all available ELISA data of NHPs (*N* = 108) vaccinated with either Ad26.ZEBOV or Ad26.Filo and MVA-BN-Filo in different vaccine sequences and intervals between doses (Supplementary Table 6)^33^. Results from the pre-specified sensitivity analyses are described in Section 1 (Supplementary Notes) in the Supplementary Appendix.

### Reporting summary

Further information on research design is available in the Nature Research Reporting Summary linked to this article.

## Supplementary information

REPORTING SUMMARY

Supplemental Information

## Data Availability

Janssen has an agreement with the Yale Open Data Access (YODA) Project to serve as the independent review panel for the evaluation of requests for clinical study reports and participant-level data from investigators and physicians for scientific research that will advance medical knowledge and public health. Data will be made available following publication and approval by YODA of any formal requests with a defined analysis plan. For more information on this process or to make a request, please visit the Yoda Project site at http://yoda.yale.edu. The data sharing policy of Janssen Pharmaceutical Companies of Johnson & Johnson is available at https://www.janssen.com/clinical-trials/transparency.
