# Supplementary material for: Non-human primate to human immunobridging demonstrates a protective effect of Ad26.ZEBOV, MVA-BN-Filo vaccine against Ebola
Source: NPJ Vaccines. 2022 Nov 30;7:156. doi: 10.1038/s41541-022-00564-z (PMC9712521; doi:10.1038/s41541-022-00564-z)
Supplement: Supplementary file 1 — REPORTING SUMMARY [file 41541_2022_564_MOESM1_ESM.pdf]

nature portfolio

Corresponding author(s): Kerstin Luhn

Last updated by author(s): Jun 7, 2022

Reporting Summary

Nature Portfolio wishes to improve the reproducibility of the work that we publish. This form provides structure for consistency and transparency in reporting. For further information on Nature Portfolio policies, see our Editorial Policies and the Editorial Policy Checklist.

Statistics

For all statistical analyses, confirm that the following items are present in the figure legend, table legend, main text, or Methods section.

n/a	Confirmed	
☐	☒	The exact sample size (*n*) for each experimental group/condition, given as a discrete number and unit of measurement
☒	☐	A statement on whether measurements were taken from distinct samples or whether the same sample was measured repeatedly
☐	☒	The statistical test(s) used AND whether they are one- or two-sided<br>*Only common tests should be described solely by name; describe more complex techniques in the Methods section.*
☒	☐	A description of all covariates tested
☐	☒	A description of any assumptions or corrections, such as tests of normality and adjustment for multiple comparisons
☐	☒	A full description of the statistical parameters including central tendency (e.g. means) or other basic estimates (e.g. regression coefficient) AND variation (e.g. standard deviation) or associated estimates of uncertainty (e.g. confidence intervals)
☒	☐	For null hypothesis testing, the test statistic (e.g. *F*, *t*, *r*) with confidence intervals, effect sizes, degrees of freedom and *P* value noted<br>*Give P values as exact values whenever suitable.*
☒	☐	For Bayesian analysis, information on the choice of priors and Markov chain Monte Carlo settings
☒	☐	For hierarchical and complex designs, identification of the appropriate level for tests and full reporting of outcomes
☒	☐	Estimates of effect sizes (e.g. Cohen's *d*, Pearson's *r*), indicating how they were calculated

Our web collection on statistics for biologists contains articles on many of the points above.

Software and code

Policy information about availability of computer code

Data collection	N/A
Data analysis	N/A

For manuscripts utilizing custom algorithms or software that are central to the research but not yet described in published literature, software must be made available to editors and reviewers. We strongly encourage code deposition in a community repository (e.g. GitHub). See the Nature Portfolio guidelines for submitting code & software for further information.

Data

Policy information about availability of data

All manuscripts must include a data availability statement. This statement should provide the following information, where applicable:

- Accession codes, unique identifiers, or web links for publicly available datasets
- A description of any restrictions on data availability
- For clinical datasets or third party data, please ensure that the statement adheres to our policy

Janssen has an agreement with the Yale Open Data Access (YODA) Project to serve as the independent review panel for the evaluation of requests for clinical study reports and participant-level data from investigators and physicians for scientific research that will advance medical knowledge and public health. Data will be made available following publication and approval by YODA of any formal requests with a defined analysis plan. For more information on this process or to make a

request, please visit the Yoda Project site at http://yoda.yale.edu. The data sharing policy of Janssen Pharmaceutical Companies of Johnson & Johnson is available at https://www.janssen.com/clinical-trials/transparency.

Human research participants

Policy information about studies involving human research participants and Sex and Gender in Research.

Reporting on sex and gender	Demographic subanalyses were conducted, including an analysis stratified by sex (female and male)
Population characteristics	The study used pooled data from five clinical studies of healthy adult participants who were vaccinated with Ad26.ZEBOV, MVA-BN-Filo. Additional information about the study populations can be found in the published primary manuscripts and on ClinicalTrials.gov; all such sources are cited in the manuscript.
Recruitment	N/A
Ethics oversight	N/A

Note that full information on the approval of the study protocol must also be provided in the manuscript.

Field-specific reporting

Please select the one below that is the best fit for your research. If you are not sure, read the appropriate sections before making your selection.

☒ Life sciences ☐ Behavioural & social sciences ☐ Ecological, evolutionary & environmental sciences

For a reference copy of the document with all sections, see nature.com/documents/nr-reporting-summary-flat.pdf

Life sciences study design

All studies must disclose on these points even when the disclosure is negative.

Sample size	N/A
Data exclusions	Several pre-specified subgroup sensitivity analyses were performed to investigate the robustness of the primary immunobridging analysis. First, EBL3001 was the only study performed in a country previously affected by an EBOV outbreak (Sierra Leone). Although previous EVD diagnosis was a study exclusion criterion and no active cases of EVD were reported in the area at the time of the study conduct, a sensitivity analysis excluding the participants of EBL3001 was pre-planned to assess the possible effect of pre-existing immunity to EBOV on the immunobridging result.
Replication	Pre-specified sensitivity immunobridging analyses were conducted to evaluate the robustness of the primary analysis.
Randomization	N/A
Blinding	N/A

Reporting for specific materials, systems and methods

We require information from authors about some types of materials, experimental systems and methods used in many studies. Here, indicate whether each material, system or method listed is relevant to your study. If you are not sure if a list item applies to your research, read the appropriate section before selecting a response.

Materials & experimental systems

n/a	Involved in the study	
☒	☐	Antibodies
☒	☐	Eukaryotic cell lines
☒	☐	Palaeontology and archaeology
☒	☐	Animals and other organisms
☐	☒	Clinical data
☒	☐	Dual use research of concern

Methods

n/a	Involved in the study	
☒	☐	ChIP-seq
☒	☐	Flow cytometry
☒	☐	MRI-based neuroimaging

Clinical data

Policy information about clinical studies

All manuscripts should comply with the ICMJE guidelines for publication of clinical research and a completed CONSORT checklist must be included with all submissions.

Clinical trial registration	NCT02416453, NCT02564523, NCT02509494, NCT02543567, NCT02543268
Study protocol	The post-hoc analysis reported in the manuscript does not have a protocol.
Data collection	For each of the clinical trials included in the current analysis, the country (or countries) in which the trial took place are stated. Specifically: EBL2001 [UK, France], EBL2002 [Kenya, Uganda, Burkina Faso, Côte d'Ivoire], EBL3001 [Sierra Leone], EBL3002 [USA], and EBL3003 [USA]. Additional information can be found in the published primary manuscripts and on ClinicalTrials.gov; all such sources are cited in the manuscript.
Outcomes	The primary analysis aimed to evaluate whether the lower limit of the CI was above a pre-defined success criterion of 20%, a cutoff agreed upon with the EMA. Immunogenicity data from healthy adult participants (aged 18-50 years) vaccinated with Ad26.ZEBOV, MVA-BN-Filo in the five contributing clinical studies were pooled. Sensitivity analyses were conducted to evaluate the robustness of the primary analysis and assess potential influencing factors, such as baseline positivity in the ELISA, sex, age, race, and geographic region.
