# Supplementary material for: Non-human primate to human immunobridging demonstrates a protective effect of Ad26.ZEBOV, MVA-BN-Filo vaccine against Ebola
Source: NPJ Vaccines. 2022 Nov 30;7:156. doi: 10.1038/s41541-022-00564-z (PMC9712521; doi:10.1038/s41541-022-00564-z)
Supplement: Supplementary file 2 — Supplemental Information [file 41541_2022_564_MOESM2_ESM.pdf]

SUPPLEMENTARY APPENDIX

1. **Supplementary Notes**
2. **Supplementary Tables and Figures**
3. **Supplementary References**

1. Supplementary Notes

RESULTS

Pre-specified sensitivity analysis excluding the participants of study EBL3001

Several pre-specified subgroup sensitivity analyses were performed to investigate the robustness of the primary immunobridging analysis. First, EBL3001 was the only study performed in a country previously affected by an EBOV outbreak (Sierra Leone).[1] Although previous EVD diagnosis was a study exclusion criterion and no active cases of EVD were reported in the area at the time of the study conduct, a sensitivity analysis excluding the participants of EBL3001 was pre-planned to assess the possible effect of pre-existing immunity to EBOV on the immunobridging result. However, in line with the lower EBOV GP-specific binding antibody responses in study EBL3001 in Sierra Leone (Supplementary Table 2), excluding EBL3001 resulted in a higher mean predicted survival probability of 62.2% in comparison to the primary analysis result of 53.4%, demonstrating that the primary immunobridging result was not driven upwards by inclusion of potentially pre-exposed participants (Fig. 2).

Pre-specified sensitivity analysis by baseline FANG ELISA level

Across the clinical studies, 4.4% to 31.4% of participants tested positive in the EBOV GP FANG ELISA prior to vaccination, except for study EBL3001, where 59.3% of baseline positivity was observed (Supplementary Table 7).[2-5] Since it is currently unclear whether the observed positivity at baseline is truly reflective of previous natural EBOV infection or due to assay cross-reactivity, since it was also observed in countries that never experienced an Ebola outbreak (including France, UK, and USA), a pre-specified sensitivity analysis stratified per baseline EBOV GP FANG ELISA level (<LLOQ, LLOQ-100, >100-1000, >1000 EU/mL) was performed to assess the potential impact of baseline positivity on the primary immunobridging

result. Most participants (N=565/764; Supplementary Table 1) tested negative prior to vaccination (<LLOQ), and this subgroup displayed a mean predicted survival probability of 57.0%, consistent with the primary immunobridging result (53.4%; Fig. 2). The mean predicted survival probability was 40.9% and 41.5% for the subgroups with baseline ELISA values between LLOQ to 100 EU/mL (N=87/756) and 100 to 1000 EU/mL (N=90/756; Fig. 2 and Supplementary Table 1), respectively, while a very small subgroup with a baseline ELISA value above 1000 EU/mL (N=14/756) had a mean predicted survival probability of 59.6%, consistent with the primary result (53.4%; Fig. 2 and Supplementary Table 1). Breakdown of the distribution of participants per study showed that this analysis was disproportionately influenced by the large number of EBL3001 study participants (59/87, 58/90, and 11/14) in all strata ≥LLOQ. In the EBL3001 study, performed in Sierra Leone, EBOV GP-specific antibody responses at baseline were overall lower compared to the four other clinical studies (Supplementary Table 8). A post-hoc subanalysis restricted to the EBL3001 participants demonstrated that the strata with baseline ELISA values <LLOQ, LLOQ to 100 EU/mL, and >100 to 1000 EU/mL showed a mean predicted survival probability fully consistent with the overall EBL3001 result (point estimates of 29.3%, 31.9%, and 28.6% in comparison to 30.9%, respectively) (Supplementary Fig. 1). Five percent of the Sierra Leonean participants (N=11/215) had a baseline ELISA value >1000 EU/mL and, in line with the observed higher antibody GMC post-vaccination, this small subgroup tended to have a higher mean predicted survival probability (52.9%); however, the 95% CI overlap across all strata. This sensitivity analysis indicates that the observed baseline positivity did not impact the immunobridging result. Although a minor percentage of participants started with a higher baseline ELISA value, the vaccine protective effect in this subgroup did not appear to be fundamentally different from the groups with no or lower baseline positivity.

Pre-specified sensitivity analysis for healthy adults using the logistic model based on all available NHP data

The pre-specified sensitivity analyses for the healthy adults also included an immunobridging analysis using a logistic model based on the NHP dataset that contained all available NHP data, including data from NHPs vaccinated with other vaccine regimens and/or intervals, with the logistic model being defined as penalized logistic regression models using Firth's method. The outcome of this sensitivity analysis was consistent with the primary immunobridging analysis result (Supplementary Table 6).

Immunobridging analyses based on the FAS

For the healthy adults, the immunobridging analysis based on the FAS (N=899) demonstrated a mean predicted survival probability of 55.1% (95% CI, 39.1-68.7), consistent with the primary immunobridging analysis based on the PPI set. The corresponding pre-specified sensitivity analyses are summarized in Supplementary Fig. 2.

For the specific subpopulations of healthy adults aged >50 years, PLWH, and children aged ≥1 year, separate immunobridging analyses were also performed on the FAS (Supplementary Table 4), with no apparent differences between PPI and FAS.

2. Supplementary Tables and Figures

Supplementary Figure 1. Mean Predicted Survival Probability and 95% CI – Subgroup Analysis Restricted to Study EBL3001 (Sierra Leone) by Baseline EBOV GP ELISA Level; PPI Analysis Set.

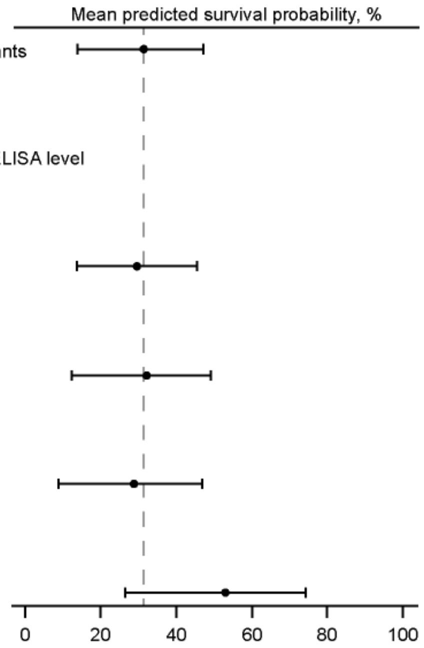

	N	GMC, EU/mL (95% CI)	Mean predicted survival probability, % (95% CI)
All EBL3001 participants	215	3918 (3461 to 4436)	30.9 (13.4 to 46.9)
Baseline EBOV GP ELISA level			
<LLOQ	84	3663 (2960 to 4532)	29.3 (13.3 to 45.2)
LLOQ–100	59	4090 (3225 to 5185)	31.9 (11.9 to 49.0)
>100–1000	58	3759 (3011 to 4692)	28.6 (8.3 to 46.7)
>1000	11	7075 (3930 to 12,738)	52.9 (26.2 to 74.2)

Immunobridging analysis was based on the EBL3001 clinical studies in healthy adults (aged ≥18 years) using a logistic regression model based on NHP data from the Ad26.ZEBOV, MVA-BN-Filo vaccine regimen in a 56-day interval. Mean predicted survival probability and the 95% bootstrapped CI are reported.

CI = confidence interval; EBOV = Ebola virus; EU = enzyme-linked immunosorbent assay units; GMC = geometric mean concentration; GP = glycoprotein; LLOQ = lower limit of quantification (36.11 EU/mL); N = number of participants with data; NHP = non-human primate; PPI = per-protocol immunogenicity; vertical dashed line = mean predicted survival probability from post-hoc subanalysis including all EBL3001 (Sierra Leone) participants.

Supplementary Figure 2. Forest Plot of Mean Predicted Survival Probability and 95% CI – Pre-specified Subgroup Analyses by Baseline EBOV GP Binding Antibody Concentration, Sex, Age Category, Race, and Region; FAS.

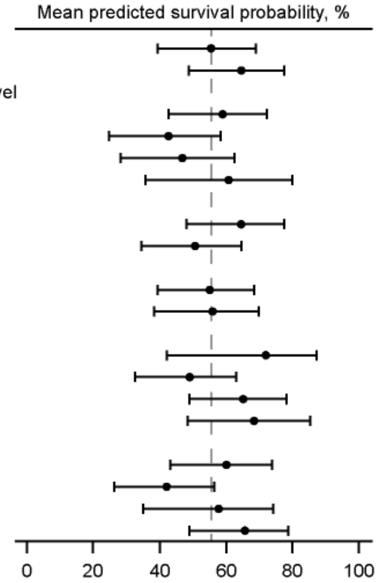

	N	GMC, EU/mL (95% CI)	Mean predicted survival probability, % (95% CI)
All participants	899	8223 (7701 to 8782)	55.1 (39.1 to 68.7)
Excluding EBL3001	635	10,923 (10,214 to 11,681)	64.2 (48.4 to 77.2)
Baseline EBOV GP ELISA level			
<LLOQ	642	9079 (8417 to 9794)	58.6 (42.5 to 71.9)
LLOQ–100	117	5640 (4666 to 6818)	42.4 (24.3 to 57.9)
>100–1000	113	6542 (5432 to 7878)	46.5 (28.0 to 62.2)
>1000	16	10,085 (5751 to 17,687)	60.6 (35.4 to 79.5)
Sex			
Female	297	11,093 (9964 to 12,349)	64.4 (47.9 to 77.2)
Male	602	7095 (6548 to 7687)	50.5 (34.3 to 64.3)
Age category			
18–30 years	520	8224 (7525 to 8988)	54.7 (39.1 to 68.1)
31–50 years	379	8222 (7458 to 9065)	55.6 (38.0 to 69.5)
Race			
Asian	9	12,435 (8693 to 17,789)	71.6 (41.9 to 86.9)
Black or African American	555	6751 (6185 to 7369)	48.7 (32.3 to 62.8)
White	317	11,171 (10,201 to 12,232)	65.0 (48.6 to 78.1)
Other	17	12,602 (7082 to 22,426)	68.1 (48.2 to 85.1)
Region			
East Africa	84	9943 (7986 to 12,379)	59.9 (42.9 to 73.5)
West Africa	353	5400 (4824 to 6044)	41.8 (25.8 to 56.2)
Europe	52	9395 (7530 to 11,722)	57.6 (34.7 to 73.8)
North America	410	11,171 (10,318 to 12,095)	65.3 (48.8 to 78.3)

This analysis was based on the pooled data of five clinical studies (EBL2001, EBL2002, EBL3001, EBL3002, and EBL3003) in healthy adults (aged 18-50 years) using a logistic regression model based on NHP data from the Ad26.ZEBOV, MVA-BN-Filo vaccine regimen in a 56-day interval. Mean predicted survival probability and the 95% bootstrapped CI are reported.

CI = confidence interval; EBOV = Ebola virus; EU = enzyme-linked immunosorbent assay units; FAS = full analysis set; GMC = geometric mean concentration; GP = glycoprotein; LLOQ = lower limit of quantification (36.11 EU/mL); N = number of participants with data; NHP = non-human primate; vertical dashed line = mean predicted survival probability from primary analysis including all participants.

Supplementary Table 1. Summary of Baseline and Demographic Characteristics for Adults (18-50 Years of Age) in the Primary Immunobridging Analysis; PPI Analysis Set

	N=764
**Study, No. (%)**	
VAC52150EBL2001	45 (5.9)
VAC52150EBL2002	115 (15.1)
VAC52150EBL3001	215 (28.1)
VAC52150EBL3002	135 (17.7)
VAC52150EBL3003	254 (33.2)
**Country, No. (%)**	
Burkina Faso	35 (4.6)
Côte d'Ivoire	17 (2.2)
France	14 (1.8)
Kenya	15 (2.0)
Sierra Leone	215 (28.1)
Uganda	48 (6.3)
UK	31 (4.1)
USA	389 (50.9)
**Age, Years**	
Mean (SD)	30.6 (9.41)
Median	28.0
Q1; Q3	22.0; 38.0
Min; Max	18; 50
**Height, cm**	

Mean (SD)	170.62 (9.198)
Median	170.20
Q1; Q3	165.00; 176.85
Min; Max	139.0; 198.1
**Weight, kg**	
Mean (SD)	74.39 (17.163)
Median	70.60
Q1; Q3	61.40; 85.95
Min; Max	43.0; 131.5
**BMI, kg/m^2**	
Mean (SD)	25.48 (5.121)
Median	24.30
Q1; Q3	21.60; 28.60
Min; Max	15.4; 41.5
**Sex, No. (%)**	
Female	267 (34.9)
Male	497 (65.1)
**Race, No. (%)**	
Asian	8 (1.0)
Black or African American	444 (58.1)
White	297 (38.9)
Other	14 (1.8)
Not Reported	1 (0.1)
**Ethnicity, No. (%)**	
Hispanic or Latino	59 (7.7)

Not Hispanic or Latino	702 (91.9)
Unknown	2 (0.3)
Not Reported	1 (0.1)
**Region, No. (%)**	
East Africa	63 (8.2)
West Africa	267 (34.9)
Europe	45 (5.9)
North America	389 (50.9)
**Baseline EBOV GP ELISA Level (EU/mL), No. (%)**	
<LLOQ	565 (74.7)*
LLOQ to 100	87 (11.5)*
>100 to 1000	90 (11.9)*
>1000	14 (1.9)*

BMI = body mass index; EBOV = Ebola virus; EU = enzyme-linked immunosorbent assay units; GP = glycoprotein; LLOQ = lower limit of quantification (36.11 EU/mL); max = maximum; min = minimum; N = number of participants with data; PPI = per-protocol immunogenicity; Q1 = 25% percentile; Q3 = 75% percentile; SD = standard deviation; UK = United Kingdom; USA = United States of America.

*N=756 (total includes only participants with data at both baseline and 21 days post-Dose 2).

Supplementary Table 2. Overview of EBOV GP-specific Antibody Responses 21 Days Post-Dose 2 (ELISA, EU/mL) for the Ad26.ZEBOV, MVA-BN-Filo Regimen With a 56-day Interval in the Five Phase 2/3 Clinical Studies – Healthy Adults (Aged 18-50 years)

	Analysis Set	Studies (Countries)					Overall (All Studies)
		**EBL2001 (FRA, UK)**	**EBL2002 (BFA, CIV, KEN, UGA)**	**EBL3001 (SL)**	**EBL3002 (USA)**	**EBL3003 (USA)**	
N	PPI	45	115	215	135	254	764
GMC		8927	8109	3918	11,054	11,052	7781
(95% CI)		(7136 to 11,168)	(6872 to 9568)	(3461 to 4436)	(9673 to 12,633)	(9959 to 12,265)	(7265 to 8333)
No./N* (% Resp)		45/45 (100.0)	113/113 (100.0)	208/212 (98.1)	135/135 (100.0)	251/251 (100.0)	752/756 (99.5)
N	FAS	52	173	264	144	266	899
GMC		9395	10836	4155	11274	11116	8223
(95% CI)		(7530 to 11,722)	(9367 to 12,535)	(3683 to 4687)	(9928 to 12,803)	(10,040 to 12,308)	(7701 to 8782)
No./N* (% Resp)		52/52 (100.0)	169/169 (100.0)	255/260 (98.1)	144/144 (100.0)	263/263 (100.0)	883/888 (99.4)

The immunogenicity data based on the primary PPI analysis set demonstrated no notable differences compared to the FAS set.

BFA = Burkina Faso; CI = confidence interval; CIV = Côte d'Ivoire; EBOV = Ebola virus; EU = enzyme-linked immunosorbent assay units; FAS = full analysis set; FRA = France; GMC = geometric mean concentration; GP = glycoprotein; KEN = Kenya; LLOQ = lower limit of quantification (36.11 EU/mL); N = number of participants with data at 21 days post-Dose 2; N* = number of participants with data at both baseline and 21 days post-Dose 2; PPI = per-protocol immunogenicity analysis set; SL = Sierra Leone; UGA = Uganda; UK = United Kingdom; USA = United States of America; % resp = percentage of participants with post-vaccination antibody concentrations >2.5-fold the LLOQ in baseline negative individuals or >2.5-fold the baseline value in baseline positive individuals.

Supplementary Table 3. Overview of EBOV GP-specific Antibody Responses 21 Days Post-Dose 2 (ELISA, EU/mL) for the Ad26.ZEBOV, MVA-BN-Filo Regimen With a 56-day Interval for Healthy Adults Aged >50 Years, PLWH Aged 18 to 50 Years, and Children (Aged 1-17 Years)

	**Analysis Set**	**Healthy Older Adults Aged >50 Years (BFA, CIV, FRA, KEN, SL, UGA, UK)**	**PLWH Aged 18 to 50 Years (BFA, CIV, KEN, UGA)**	**Children Aged 1 to 17 Years (BFA, CIV, KEN, SL, UGA)**
N	PPI	53	59	487
GMC		7700	5283	13,509
(95% CI)		(6066 to 9775)	(4094 to 6817)	(12,206 to 14,951)
No./N* (% Resp)		50/51 (98.0)	58/58 (100.0)	471/478 (98.5)
N	FAS	69	59	532
GMC		8901	5283	13,450

	Analysis Set	Healthy Older Adults Aged >50 Years (BFA, CIV, FRA, KEN, SL, UGA, UK)	PLWH Aged 18 to 50 Years (BFA, CIV, KEN, UGA)	Children Aged 1 to 17 Years (BFA, CIV, KEN, SL, UGA)
(95% CI)		(7217 to 10977)	(4094 to 6817)	(12,194 to 14,836)
No./N* (% Resp)		66/67 (98.5)	58/58 (100.0)	514/522 (98.5)

The immunogenicity data based on the PPI demonstrated no notable differences compared to the FAS analysis set. Studies including older adults (>50 years): EBL2001, EBL2002, and EBL3001; study including PLWH: EBL2002; studies including children: EBL2002 and EBL3001.

BFA = Burkina Faso; CI = confidence interval; CIV = Côte d'Ivoire; EBOV = Ebola virus; EU = enzyme-linked immunosorbent assay units; FAS = full analysis set; FRA = France; GMC = geometric mean concentration; GP = glycoprotein; KEN = Kenya; LLOQ = lower limit of quantification (36.11 EU/mL); N = number of participants with data at 21 days post–Dose 2; N* = number of participants with data at both baseline and 21 days post-Dose 2; PLWH = people living with human immunodeficiency virus; PPI = per-protocol immunogenicity analysis set; SL = Sierra Leone; UGA = Uganda; UK = United Kingdom; USA = United States of America; % resp = percentage of participants with post-vaccination antibody concentrations >2.5-fold the LLOQ in baseline negative individuals or >2.5-fold the baseline value in baseline positive individuals.

Supplementary Table 4. Immunobridging Analysis for Healthy Adults Aged >50 Years, PLWH Aged 18 to 50 Years, and Children (Aged 1-17 Years) Using a Logistic Regression Model Based on Data From NHPs Vaccinated With the Ad26.ZEBOV, MVA-BN-Filo Vaccine Regimen in a 56-day Interval Based on the FAS

**Elderly Healthy Participants (Aged >50 Years)**	
No. Participants Vaccinated	69
Mean Predicted Survival Probability, % (95% CI)	57.8 (36.2 to 73.6)
**PLWH (Aged 18-50 Years)**	
No. Participants Vaccinated	59
Mean Predicted Survival Probability, % (95% CI)	42.0 (22.4 to 58.9)
**Children (Aged 1-17 Years)**	
No. Participants Vaccinated	532
Mean Predicted Survival Probability, % (95% CI)	69.8 (59.5 to 79.7)
Children (Aged 12-17 Years)	

No. Participants Vaccinated	194
Mean Predicted Survival Probability, % (95% CI)	63.7 (49.7 to 76.0)
Children (Aged 4-11 Years)	
No. Participants Vaccinated	196
Mean Predicted Survival Probability, % (95% CI)	66.7 (55.3 to 77.6)
Children (Aged 1-3 Years)	
No. Participants Vaccinated	142
Mean Predicted Survival Probability, % (95% CI)	82.6 (75.1 to 89.8)

This analysis was based on the pooled data of participants vaccinated with Ad26.ZEBOV, MVA-BN-Filo in a 56-day interval in five clinical studies (EBL2001, EBL2002, EBL3001, EBL3002, and EBL3003) using a logistic regression model based on data from NHPs vaccinated with the Ad26.ZEBOV, MVA-BN-Filo vaccine regimen in a 56-day interval.

CI = confidence interval; FAS = full analysis set; NHP = non-human primate; PLWH = people living with human immunodeficiency virus.

Supplementary Table 5. NHP Immunogenicity Data Used to Construct the Logistic Regression Models

Study	NHP ID	Vaccine regimen	Survival	Log10-transformed ELISpot values	Log10-transformed neutralizing antibody levels	Interval	EIA EU/mL (Q² Solutions)
C25 study 1	33375	Negative control	0	2.176091259	1.612711821	8 wks interval	13.62
C25 study 1	33376	Negative control	0	2.176091259	1.719853949	8 wks interval	65.26
C25 study 1	33368	Ad26.Filo, Ad26.Filo, MVA-BN-Filo	1	2.787106093	3.717161306	4 wks interval	38179.84
C25 study 1	33378	Ad26.Filo, Ad26.Filo, MVA-BN-Filo	1	2.761551989	3.70737418	4 wks interval	48517.12
C25 study 1	33377	Ad26.Filo, Ad35.Filo	1	2.846646329	3.882220733	8 wks interval	33186.56
C25 study 1	33379	Ad26.Filo, Ad35.Filo	1	2.917768002	4.033223785	8 wks interval	46296.32
C25 study 1	33370	Ad26.Filo, MVA-BN-Filo	1	3.260667537	4.329461544	8 wks interval	137661.44
C25 study 1	33373	Ad26.Filo, MVA-BN-Filo	1	2.790636962	3.595020254	8 wks interval	26231.04
C25 study 1	33369	Ad26.ZEBOV, Ad35.ZEBOV	1	2.703291378	3.604767719	4 wks interval	50081.28
C25 study 1	33381	Ad26.ZEBOV, Ad35.ZEBOV	1	2.996730515	4.059543297	4 wks interval	1854.96
C25 study 1	33367	MVA-BN-Filo, Ad26.Filo	1	3.049218023	3.676326078	8 wks interval	53881.60
C25 study 1	33380	MVA-BN-Filo, Ad26.Filo	1	3.051152522	3.826830847	8 wks interval	33576.96
C29 study 1	34187	Ad26.ZEBOV, MVA-BN-Filo High	1	2.806179974		4 wks interval	20382.72
C29 study 1	34188	Ad26.ZEBOV, MVA-BN-Filo Standard	1	3.128722284	3.62951451	8 wks interval	18321.92
C29 study 1	34189	Ad26.ZEBOV, MVA-BN-Filo High	1	3.16879202		8 wks interval	93045.76
C29 study 1	34190	MVA-BN-Filo, Ad26.ZEBOV, Standard	1	2.750122527		4 wks interval	16321.28
C29 study 1	34191	Ad26.ZEBOV, MVA-BN-Filo Standard	1	3.070960916	3.322506765	4 wks interval	14128.64
C29 study 1	34192	Negative control	0	2.176091259	1.486421977	8 wks interval	13.62
C29 study 1	34193	Ad26.ZEBOV, MVA-BN-Filo High	1	3.331427297	3.765026397	8 wks interval	
C29 study 1	34194	MVA-BN-Filo, Ad26.ZEBOV, Standard	1	2.966141733	2.468064504	4 wks interval	11255.04
C29 study 1	34195	Ad26.ZEBOV, MVA-BN-Filo High	0	3.239924813	2.812390753	4 wks interval	3935.36
C29 study 1	34196	Ad26.ZEBOV, MVA-BN-Filo Standard	1	2.849726444	3.507578563	8 wks interval	10766.08
C29 study 1	34197	Ad26.ZEBOV, MVA-BN-Filo High	1	2.70969387	3.017056348	4 wks interval	10600.32
C29 study 1	34198	Ad26.ZEBOV, MVA-BN-Filo High	1	3.202079441	3.864612695	8 wks interval	42483.20
C29 study 1	34199	Ad26.ZEBOV, MVA-BN-Filo Standard	1	2.924279286	3.06867111	4 wks interval	18609.92
C29 study 1	34200	Ad26.ZEBOV, MVA-BN-Filo Standard	1	2.730378469	3.786337055	8 wks interval	22927.36

Study	NHP ID	Vaccine regimen	Survival	Log10-transformed ELISpot values	Log10-transformed neutralizing antibody levels	Interval	EIA EU/mL (Q² Solutions)
C29 study 1	34201	Ad26.ZEBOV, MVA-BN-Filo High	1	3.193124598	4.267634548	8 wks interval	84992.00
C29 study 1	34202	Negative control	0	2.176091259	1.653890449	8 wks interval	14.95
C29 study 1	34203	Ad26.ZEBOV, MVA-BN-Filo High	0	2.515211304	2.191784293	4 wks interval	11066.88
C29 study 1	34204	Ad26.ZEBOV, MVA-BN-Filo Standard	1	3.325310372	3.521504241	8 wks interval	
C29 study 1	34205	MVA-BN-Filo, Ad26.ZEBOV, Standard	0	2.511883361	3.253652174	4 wks interval	12994.56
C29 study 1	34206	MVA-BN-Filo, Ad26.ZEBOV, Standard	0	2.72427587	2.958800268	4 wks interval	19605.12
C29 study 2	AE868G	MVA-BN-Filo, Ad26.Filo	0	2.612783857	2.89740945	4 wks interval	11706.88
C29 study 2	AF805H	Negative control	0	2.176091259	1.602059991	8 wks interval	13.62
C29 study 2	AG556G	Ad26.Filo, MVA-BN-Filo	1	2.877946952	3.023466837	4 wks interval	15871.36
C29 study 2	AH258I	MVA-BN-Filo, Ad26.Filo	1	2.799340549	2.243952826	4 wks interval	14274.56
C29 study 2	AH649H	MVA-BN-Filo, Ad26.Filo	1	2.384711743	3.866968847	8 wks interval	27779.84
C29 study 2	AJ388H	MVA-BN-Filo, Ad26.Filo	0	2.669781615	2.727248945	4 wks interval	8709.76
C29 study 2	AK65E	Negative control	0	2.176091259	1.78503071	8 wks interval	26.41
C29 study 2	AK681G	MVA-BN-Filo, Ad26.Filo	1	2.730378469	3.543247321	8 wks interval	22190.72
C29 study 2	AL599B	Ad26.Filo, MVA-BN-Filo	1	2.912487761	3.804277376	8 wks interval	71605.76
C29 study 2	AL655C	MVA-BN-Filo, Ad26.Filo	1	2.568201724	3.533695154	8 wks interval	15663.36
C29 study 2	AL852D	Ad26.Filo, MVA-BN-Filo	0	2.505149978	3.271348228	4 wks interval	38620.16
C29 study 2	AP10B	Ad26.Filo, MVA-BN-Filo	0	2.393575203	3.308047627	8 wks interval	36921.60
C29 study 2	BF318D	MVA-BN-Filo, Ad26.Filo	0	2.46612587	3.094283639	8 wks interval	8479.36
C29 study 2	F057	Ad26.Filo, MVA-BN-Filo	1	2.943247125	3.312513233	8 wks interval	21986.56
C29 study 2	F299	Ad26.Filo, MVA-BN-Filo	1	2.772688355	3.576253665	4 wks interval	38686.72
C29 study 2	F364	MVA-BN-Filo, Ad26.Filo	0	2.278753601	2.770349338	4 wks interval	8381.12
C29 study 2	GA707C	Ad26.Filo, MVA-BN-Filo	1	2.384711743	3.586925079	4 wks interval	37102.08
C29 study 2	GA951A	Ad26.Filo, MVA-BN-Filo	1	2.807873132	3.68257919	8 wks interval	24189.44
Study 12	33649	Ad26.ZEBOV, Ad35.ZEBOV	0	2.565257343	3.151701773	4 wks interval	15683.20
Study 12	33650	Ad26.ZEBOV, MVA-BN-Filo	1	3.256477206	3.735457731	6 wks interval	15290.88
Study 12	33651	Negative control	0	2.176091259	1.756295232	4 wks interval	13.62
Study 12	33653	Ad26.ZEBOV, MVA-BN-Filo	1	3.248586244	3.215538825	4 wks interval	15482.24
Study 12	33656	Ad26.ZEBOV, MVA-BN-Filo	1	2.944482672	3.836061136	6 wks interval	26946.56
Study 12	33657	Ad26.ZEBOV, MVA-BN-Filo	1	2.176091259	3.682650505	6 wks interval	18748.16

Study	NHP ID	Vaccine regimen	Survival	Log10-transformed ELISpot values	Log10-transformed neutralizing antibody levels	Interval	EIA EU/mL (Q² Solutions)
Study 12	33659	Negative control	0	2.176091259	1.639352993	4 wks interval	13.62
Study 12	33660	Ad26.ZEBOV, MVA-BN-Filo	0	2.890700398	3.446599775	6 wks interval	13573.12
Study 12	33661	Ad26.ZEBOV, MVA-BN-Filo	1	2.317018101	3.308632252	6 wks interval	11045.76
Study 12	33663	Ad26.ZEBOV, MVA-BN-Filo	1	2.511883361	3.296823591	4 wks interval	11267.20
Study 12	33664	Ad26.ZEBOV, Ad35.ZEBOV	0	2.176091259	3.519731861	4 wks interval	29786.24
Study 12	33665	Ad26.ZEBOV, Ad35.ZEBOV	1	3.499687083	3.666426292	4 wks interval	24327.68
Study 12	33666	Ad26.ZEBOV, MVA-BN-Filo	0	2.967313918	2.185020291	4 wks interval	2364.72
Study 12	33668	Ad26.ZEBOV, Ad35.ZEBOV	1	2.505149978	3.921773487	4 wks interval	49247.36
Study 12	33671	Ad26.ZEBOV, MVA-BN-Filo	0	2.662757832	3.760051856	4 wks interval	31722.88
Study 12	33672	Ad26.ZEBOV, MVA-BN-Filo	0	2.734399743	3.555083948	4 wks interval	8568.00
C29#8	35994	Ad26.ZEBOV 5x10^10, MVA-BN-Filo 1x10^8	1	2.727337888	4.764179475	8 wks interval	34311.68
C29#8	35995	Ad26.ZEBOV 5x10^9, MVA-BN-Filo 1x10^8	1	2.777245526	5.280194049	8 wks interval	42926.08
C29#8	35996	Ad26.ZEBOV 1x10^11, MVA-BN-Filo 5x10^8	1	3.40419206	4.968745204	8 wks interval	23527.68
C29#8	35997	Ad26.ZEBOV 2x10^10, MVA-BN-Filo 1x10^8	1	2.665111737	5.04305995	8 wks interval	36779.52
C29#8	35998	Ad26.ZEBOV 2x10^10, MVA-BN-Filo 1x10^8	NA*	3.053558592		8 wks interval	
C29#8	35999	Ad26.ZEBOV 2x10^9, MVA-BN-Filo 1x10^8	1	2.644684718	3.54590189	8 wks interval	12476.16
C29#8	36000	Ad26.ZEBOV 5x10^10, MVA-BN-Filo 1x10^8	1	3.170994702	4.963829352	8 wks interval	48532.48
C29#8	36001	Ad26.ZEBOV 5x10^9, MVA-BN-Filo 1x10^8	1	3.061169643	4.899346632	8 wks interval	48396.80
C29#8	36002	Ad26.ZEBOV 5x10^10, MVA-BN-Filo 1x10^8	1	2.923632533	4.911622836	8 wks interval	24368.64
C29#8	36003	Negative control	0	2	1.602059991	8 wks interval	13.62
C29#8	36004	Ad26.ZEBOV 2x10^10, MVA-BN-Filo 1x10^8	1	2.423245874	5.157637564	8 wks interval	51074.56
C29#8	36005	Ad26.ZEBOV 5x10^10, MVA-BN-Filo 1x10^8	1	3.424268947	4.707695452	8 wks interval	21962.24
C29#8	36006	Ad26.ZEBOV 5x10^10, MVA-BN-Filo 1x10^8	1	2.986211716	4.888383741	8 wks interval	56125.44
C29#8	36007	Ad26.ZEBOV 1x10^11, MVA-BN-Filo 5x10^8	1	2	4.96361881	8 wks interval	46389.76
C29#8	36008	Ad26.ZEBOV 5x10^10, MVA-BN-Filo 1x10^8	1	2.498310554	4.805240662	8 wks interval	19074.56
C29#8	36009	Ad26.ZEBOV 5x10^9, MVA-BN-Filo 1x10^8	1	2.197280558	4.762880093	8 wks interval	32757.76
C29#8	36010	Ad26.ZEBOV 5x10^9, MVA-BN-Filo 1x10^8	1	3.184691431	4.70776823	8 wks interval	28006.40
C29#8	36011	Ad26.ZEBOV 2x10^9, MVA-BN-Filo 1x10^8	1	2.751086555	4.678022736	8 wks interval	18941.44
C29#8	36012	Ad26.ZEBOV 2x10^10, MVA-BN-Filo 1x10^8	1	2.697882909	4.597990704	8 wks interval	23884.80
TO14#1	C79314	Ad26.ZEBOV 5x10^9, MVA-BN-Filo 1x10^4	0		2.630240488	8 wks interval	1374.04

Study	NHP ID	Vaccine regimen	Survival	Log10-transformed ELISpot values	Log10-transformed neutralizing antibody levels	Interval	EIA EU/mL (Q^2 Solutions)
TO14#1	C79316	Ad26.ZEBOV 5x10^8, MVA-BN-Filo 1x10^4	0		2.10430885	8 wks interval	434.49
TO14#1	C79332	Ad26.ZEBOV 5x10^7 , MVA-BN-Filo 1x10^4	0		1.602059991	8 wks interval	310.98
TO14#1	C79342	Ad26.ZEBOV 5x10^9, MVA-BN-Filo 1x10^4	0		2.583524543	8 wks interval	636.38
TO14#1	C79401	Ad26.ZEBOV 5x10^9, MVA-BN-Filo 1x10^6	0		3.787842726	8 wks interval	8225.28
TO14#1	C79427	Negative control	0		1.602059991	8 wks interval	13.62
TO14#1	C79434	Ad26.ZEBOV 5x10^9, MVA-BN-Filo 1x10^6	0		3.464841371	8 wks interval	6783.04
TO14#1	C79435	Ad26.ZEBOV 5x10^9, MVA-BN-Filo 1x10^8	1		4.305370934	8 wks interval	19376.00
TO14#1	C79438	Ad26.ZEBOV 5x10^9, MVA-BN-Filo 1x10^8	1		4.71456581	8 wks interval	17000.96
TO14#1	C79442	Ad26.ZEBOV 5x10^8, MVA-BN-Filo 1x10^4	0		2.952239564	8 wks interval	984.28
TO14#1	C79456	Negative control	0		1.602059991	8 wks interval	13.62
TO14#1	C79459	Ad26.ZEBOV 5x10^7 , MVA-BN-Filo 1x10^4	0		1.602059991	8 wks interval	13.62
TO14#1	C79714	Ad26.ZEBOV 5x10^9, MVA-BN-Filo 1x10^6	0		4.003884038	8 wks interval	5187.84
TO14#1	C79717	Ad26.ZEBOV 5x10^9, MVA-BN-Filo 1x10^6	1		3.719592225	8 wks interval	4112.32
TO14#1	C79721	Ad26.ZEBOV 5x10^9, MVA-BN-Filo 1x10^4	0		2.587507798	8 wks interval	504.21
TO14#1	C79722	Ad26.ZEBOV 5x10^9, MVA-BN-Filo 1x10^8	1		4.623746025	8 wks interval	23872.96
TO14#1	C79730	Ad26.ZEBOV 5x10^9, MVA-BN-Filo 1x10^8	1		5.093564781	8 wks interval	42112.00
TO14#1	C79740	Ad26.ZEBOV 5x10^9, MVA-BN-Filo 1x10^4	0		3.106839949	8 wks interval	1687.60
TO14#1	C79742	Ad26.ZEBOV 5x10^7 , MVA-BN-Filo 1x10^4	0		1.602059991	8 wks interval	13.62
TO14#1	C79743	Ad26.ZEBOV 5x10^9, MVA-BN-Filo 1x10^4	1		2.960086402	8 wks interval	1769.44
TO14#1	C79745	Ad26.ZEBOV 5x10^8, MVA-BN-Filo 1x10^4	0		2.566905477	8 wks interval	1728.00
TO14#1	C79746	Ad26.ZEBOV 5x10^9, MVA-BN-Filo 1x10^6	0		4.262159768	8 wks interval	9997.76
TO14#1	C79748	Ad26.ZEBOV 5x10^7 , MVA-BN-Filo 1x10^4	0		1.602059991	8 wks interval	407.96
TO14#1	C79749	Ad26.ZEBOV 5x10^8, MVA-BN-Filo 1x10^4	0		2.597862949	8 wks interval	646.15
TO14#2	C72952	Ad26.ZEBOV 5x10^9, MVA-BN-Filo 1x10^5	0		3.291119726	8 wks interval	2430.80
TO14#2	C75673	Ad26.ZEBOV 5x10^9, MVA-BN-Filo 1x10^4	0		1.942942146	8 wks interval	167.62
TO14#2	C76102	Negative control	0		1.602059991	8 wks interval	13.62
TO14#2	C76145	Ad26.ZEBOV 5x10^9, MVA-BN-Filo 1x10^4	0		2.612838617	8 wks interval	1118.20
TO14#2	C76593	Ad26.ZEBOV 5x10^9, MVA-BN-Filo 1x10^6	0		3.784442991	8 wks interval	5409.12
TO14#2	C77622	Ad26.ZEBOV 5x10^9, MVA-BN-Filo 1x10^8	1		4.827252196	8 wks interval	54090.24
TO14#2	C79138	Ad26.ZEBOV 5x10^9, MVA-BN-Filo 1x10^6	0		3.592093511	8 wks interval	2640.32

Study	NHP ID	Vaccine regimen	Survival	Log10-transformed ELISpot values	Log10-transformed neutralizing antibody levels	Interval	EIA EU/mL (Q^2 Solutions)
TO14#2	C80688	Ad26.ZEBOV 5x10^9, MVA-BN-Filo 1x10^4	0		2.144335046	8 wks interval	453.60
TO14#2	C80984	Ad26.ZEBOV 5x10^9, MVA-BN-Filo 1x10^5	0		2.721126496	8 wks interval	1122.44
TO14#2	C81174	Ad26.ZEBOV 5x10^9, MVA-BN-Filo 1x10^5	0		2.872861239	8 wks interval	975.40
TO14#2	C81261	Ad26.ZEBOV 5x10^9, MVA-BN-Filo 1x10^4	0		3.102076724	8 wks interval	983.16
TO14#2	C81876	Ad26.ZEBOV 5x10^9, MVA-BN-Filo 1x10^5	0		2.786801945	8 wks interval	526.72
TO14#2	C81947	Ad26.ZEBOV 5x10^9, MVA-BN-Filo 1x10^6	0		3.753185478	8 wks interval	9158.40
TO14#2	C82756	Ad26.ZEBOV 5x10^9, MVA-BN-Filo 1x10^5	0		2.656909947	8 wks interval	549.88
TO14#2	C82979	Ad26.ZEBOV 5x10^9, MVA-BN-Filo 1x10^8	1		5.108443493	8 wks interval	40261.12
TO14#2	C83021	Ad26.ZEBOV 5x10^9, MVA-BN-Filo 1x10^4	0		2.454647935	8 wks interval	306.16
TO14#2	C83420	Ad26.ZEBOV 5x10^9, MVA-BN-Filo 1x10^6	0		3.705153471	8 wks interval	4253.12
TO14#2	C83678	Ad26.ZEBOV 5x10^9, MVA-BN-Filo 1x10^5	0		2.85245596	8 wks interval	1305.60
TO14#2	C83733	Ad26.ZEBOV 5x10^9, MVA-BN-Filo 1x10^4	0		3.403831341	8 wks interval	1518.52
TO14#2	C83904	Ad26.ZEBOV 5x10^9, MVA-BN-Filo 1x10^5	0		3.09093289	8 wks interval	1719.20
TO14#2	C83924	Ad26.ZEBOV 5x10^9, MVA-BN-Filo 1x10^5	0		3.277086647	8 wks interval	1218.48
TO14#2	C83985	Negative control	0		1.664120886	8 wks interval	38.91
TO14#2	C84344	Ad26.ZEBOV 5x10^9, MVA-BN-Filo 1x10^4	0		2.285141943	8 wks interval	548.48

Cells shaded in gray indicate sample not analyzed.

*Study C29#8, NHP #35998 was taken out of the study just prior to challenge due to a rash in the groin area.

EIA, enzyme immunoassay; EU, enzyme-linked immunosorbent assay units; NHP, non-human primate; wks, weeks.

Supplementary Table 6. Immunobridging Sensitivity Analysis Using the Logistic Regression Model Based on NHP Data From All Vaccine Regimens Combined

Participants Vaccinated	N=764
**Sensitivity Analysis**	
Mean Predicted Survival Probability, % (95% CI)	45.6 (34.8 to 55.9)

CI = confidence interval; NHP = non-human primate; PPI = per-protocol immunogenicity.

This analysis was based on the pooled data of healthy adults (18 to 50 years of age) vaccinated with Ad26.ZEBOV, MVA-BN-Filo in a 56-day interval in five clinical studies (EBL2001, EBL2002, EBL3001, EBL3002, and EBL3003) using the logistic model based on NHP data (N=108) from all vaccine regimens combined.[6] PPI analysis set.

Supplementary Table 7. EBOV GP Binding Antibody Concentrations (ELISA, EU/mL) at Baseline for the Healthy Adult Data Set in the Five Phase 2/3 Studies

	All Participants		Positive Participants	
Study (Country)	N	GMC (95% CI)	No. (%)	Binding Antibody Concentrations Min; Max
EBL2001 (FRA, UK)	222	<LLOQ (<LLOQ to <LLOQ)	17 (7.7)	40; 694
EBL2002 (BFA, CIV, KEN, UGA)	392	<LLOQ (<LLOQ to 39)	123 (31.4)	36; 10,441
EBL3001 (SL)	297	63 (54 to 74)	176 (59.3)	37; 13,341

	All Participants		Positive Participants	
Study (Country)	N	GMC (95% CI)	No. (%)	Binding Antibody Concentrations Min; Max
EBL3002 (USA)	206	<LLOQ (<LLOQ to <LLOQ)	9 (4.4)	37; 2444
EBL3003 (USA)	301	<LLOQ (<LLOQ to <LLOQ)	25 (8.3)	37; 2717

The analysis is based on the PPI analysis set, including all healthy adults (not restricted to the 18-50 years of age of participants in the immunobridging analysis set). Data from participants receiving active vaccine and placebo/control vaccine are pooled. No. (%): number (percentage) of all participants who were found to be positive in the EBOV GP ELISA at baseline.

BFA = Burkina Faso; CI = confidence interval; CIV = Côte d'Ivoire; EBOV = Ebola virus; EU = enzyme-linked immunosorbent assay units; FAS = full analysis set; FRA = France; GMC = geometric mean concentration; GP = glycoprotein; KEN = Kenya; LLOQ = lower limit of

quantification (36.11 EU/mL); max = maximum; min = minimum; N = number of participants with data; PPI = per-protocol immunogenicity analysis set; SL = Sierra Leone; UGA = Uganda; UK = United Kingdom; USA = United States of America.

Supplementary Table 8. Number of Healthy Adult (Aged 18-50 Years) Participants in the PPI Analysis Set by Baseline EBOV GP ELISA Level

		Studies (Countries)				
		EBL2001 (FRA, UK)	EBL2002 (BFA, CIV, KEN, UGA)	EBL3001 (SL)	EBL3002 (USA)	EBL3003 (USA)
Distribution By	N	45	113	212	135	251
Baseline ELISA Level, No. (%)	<LLOQ	42 (93.3)	77 (68.1)	84 (39.6)	131 (97.0)	231 (92.0)
	LLOQ to 100 EU/mL	2 (4.4)	15 (13.3)	59 (27.8)	2 (1.5)	9 (3.6)
	>100 to 1000 EU/mL	1 (2.2)	20 (17.7)	58 (27.4)	1 (0.7)	10 (4.0)
	>1000 EU/mL	0	1 (0.9)	11 (5.2)	1 (0.7)	1 (0.4)

BFA = Burkina Faso; CI = confidence interval; CIV = Côte d'Ivoire; EBOV = Ebola virus; EU = enzyme-linked immunosorbent assay units; FRA = France; GP = glycoprotein; KEN = Kenya; LLOQ = lower limit of quantification (36.11 EU/mL); N = number of participants with data at both baseline and 21 days post-Dose 2 (7 out of 764 participants included in the PPI immunobridging analysis did not have baseline EBOV GP ELISA result); PPI = per-protocol immunogenicity analysis set; SL = Sierra Leone; UGA = Uganda; UK = United Kingdom; USA = United States of America.

3. Supplementary References

1. Janssen Vaccines & Prevention B.V. Staged phase 3 study to assess the safety and immunogenicity of Ebola candidate vaccines Ad26.ZEBOV and MVA-BN-Filo (EBOVAC-Salone). ClinicalTrials.gov. NCT02509494. https://clinicaltrials.gov/ct2/show/NCT02509494. (2022) Accessed August 1, 2022.
2. Pollard, A.J., *et al.* Safety and immunogenicity of a two-dose heterologous Ad26.ZEBOV and MVA-BN-Filo Ebola vaccine regimen in adults in Europe (EBOVAC2): a randomised, observer-blind, participant-blind, placebo-controlled, phase 2 trial. *Lancet Infect Dis* **21**, 493-506 (2021).
3. Ishola, D., *et al.* Safety and long-term immunogenicity of the two-dose heterologous Ad26.ZEBOV and MVA-BN-Filo Ebola vaccine regimen in adults in Sierra Leone: a combined open-label, non-randomised stage 1, and a randomised, double-blind, controlled stage 2 trial. *Lancet Infect Dis* **22**, 97-109 (2022).
4. Barry, H., *et al.* Safety and immunogenicity of 2-dose heterologous Ad26.ZEBOV, MVA-BN-Filo Ebola vaccination in healthy and HIV-infected adults: a randomised, placebo-controlled phase II clinical trial in Africa. *PLoS Med* **18**, e1003813 (2021).
5. Bockstal, V., *et al.* Assessments of different batches and dose levels of a two-dose Ad26.ZEBOV and MVA-BN-Filo vaccine regimen. *NPJ Vaccines* **6**, 157 (2021).
6. Roozendaal, R., *et al.* Nonhuman primate to human immunobridging to infer the protective effect of an Ebola virus vaccine candidate. *NPJ Vaccines* **5**, 112 (2020).
